# ERAP1 and ERAP2 Haplotypes Influence Suboptimal HLA-B*27:05-Restricted Anti-Viral CD8+ T Cell Responses Cross-Reactive to Self-Epitopes

**DOI:** 10.3390/ijms241713335

**Published:** 2023-08-28

**Authors:** Valentina Tedeschi, Giorgia Paldino, Josephine Alba, Emanuele Molteni, Fabiana Paladini, Rossana Scrivo, Mattia Congia, Alberto Cauli, Rosalba Caccavale, Marino Paroli, Manuela Di Franco, Loretta Tuosto, Rosa Sorrentino, Marco D’Abramo, Maria Teresa Fiorillo

**Affiliations:** 1 Department of Biology and Biotechnologies “Charles Darwin”, Sapienza University of Rome, 00185 Rome, Italy; giorgia.paldino@uniroma1.it (G.P.); loretta.tuosto@uniroma1.it (L.T.); rosa.sorrentino@uniroma1.it (R.S.); mariateresa.fiorillo@uniroma1.it (M.T.F.); 2 Department of Biology, University of Fribourg, Chemin du Musée, 1700 Fribourg, Switzerland; josephine.alba@unifr.ch; 3 Rheumatology Unit, Department of Clinical Internal, Anaesthesiological and Cardiovascular Sciences, Policlinico Umberto I, Sapienza University of Rome, 00161 Rome, Italy; emanuele.molteni@uniroma1.it (E.M.); rossana.scrivo@uniroma1.it (R.S.); manuela.difranco@uniroma1.it (M.D.F.); 4 Rheumatology Unit, AOU and University of Cagliari, 09042 Monserrato, Italy; mattiacongia@yahoo.it (M.C.); cauli@unica.it (A.C.); 5 Department of Biotechnology and Medical Surgical Sciences, Division of Clinical Immunology and Rheumatology, Sapienza University of Rome c/o Polo Pontino, 04100 Latina, Italy; rosalba_caccavale@yahoo.it (R.C.); marino.paroli@uniroma1.it (M.P.); 6 Department of Chemistry, Sapienza University of Rome, 00185 Rome, Italy

**Keywords:** human leukocyte antigen (HLA)-B*27, Endoplasmic Reticulum AminoPeptidase (ERAP)1 and ERAP2 haplotypes, ankylosing spondylitis, CD8+ T cells, peptides, antigen processing and presentation, anti-viral immunity

## Abstract

The human leukocyte antigen (HLA)-B*27 family of alleles is strongly associated with ankylosing spondylitis (AS), a chronic inflammatory disorder affecting the axial and peripheral joints, yet some HLA-B*27 variants not associated with AS have been shown. Since no major differences in the ligandome of associated compared to not-associated alleles have emerged, a plausible hypothesis is that the quantity rather than the quality of the presented epitopes makes the difference. In addition, the Endoplasmic Reticulum AminoPeptidases (ERAPs) 1 and 2, playing a crucial role in shaping the HLA class I epitopes, act as strong AS susceptibility factors, suggesting that an altered peptidome might be responsible for the activation of pathogenic CD8+ T cells. In this context, we have previously singled out a B*27:05-restricted CD8+ T cell response against pEBNA3A (RPPIFIRRL), an EBV peptide lacking the B*27 classic binding motif. Here, we show that a specific ERAP1/2 haplotype negatively correlates with such response in B*27:05 subjects. Moreover, we prove that the B*27:05 allele successfully presents peptides with the same suboptimal N-terminal RP motif, including the self-peptide, pDYNEIN (RPPIFGDFL). Overall, this study underscores the cooperation between the HLA-B*27 and ERAP1/2 allelic variants in defining CD8+ T cell reactivity to suboptimal viral and self-B*27 peptides and prompts further investigation of the B*27:05 peptidome composition.

## 1. Introduction

The human leukocyte antigen (HLA)-B*27 carriage is strongly related to the development of spondyloarthritis (SpA), a group of chronic inflammatory rheumatic diseases, whose prototype is ankylosing spondylitis (AS) [1,2,3]. However, the immunopathogenic mechanism of AS remains still unclear. One theory, the so-called arthritogenic peptide hypothesis, is strictly linked to the conventional role of HLA-B*27, as part of HLA class I molecules, that is to present microbial/self-epitopes, usually of 8–10 aa in length, which are recognized by the T cell receptor (TCR) on cross-reactive CD8+ T lymphocytes [4,5,6]. Along this line, Genome-Wide Association Studies (GWAS) identified the ERAP1 and 2 aminopeptidases, which generate or destroy HLA class I antigenic ligands by trimming the N-terminal end of the peptides, as predisposing factors for AS [7,8,9,10,11]. Notably, several single nucleotide polymorphisms (SNPs) associated with AS susceptibility allowed us to define an ERAP risk haplotype that is characterized by a high level of expression and enzymatic activity of ERAP1 together with the expression of ERAP2, thus suggesting their possible involvement in the alteration of the HLA-B*27 peptidome [10,12,13,14,15].

The HLA-B*27 peptidome consists mainly of 9mers and 10mers with Arg at P2 as the principal anchor residue, and basic, aliphatic or aromatic residues at the C-terminus [16]. However, we and other groups have disclosed Gln and Lys as alternative P2 residues [16,17,18]. Moreover, the N- and C-termini, as well as the main chain of the peptides, are anchored, via hydrogen bonds and salt bridges, to the HLA-B*27 groove made by (A–F) pockets [19]. Notably, within the HLA-B*27 family, not all subtypes are associated with AS [16] and most of the allelic variance is found in the binding groove cleft, supporting the relevance of the peptide repertoire [10]. However, there are no compelling data about the differences between the peptidome displayed by the AS-associated (HLA-B*27:05, -B*27:02, -B*27:04 and -B*27:07) and the non-AS-associated alleles (HLA-B*27:06 and -B*27:09) [20].

Apart from the axial spine and the sacroiliac joints, up to 60% of AS patients manifest a subclinical inflammation at the gut site, suggesting that the dysbiosis could be the source of several bacterial antigens that activate the T cell compartment [21,22]. Such immune cells, once triggered in the inflamed sites, could cross-react with self-peptides, increasing the inflammation. This theory has been recently strengthened by the identification of HLA-B*27-restricted self and microbial antigens recognized by public TCRs using TRBV9-CDR3β-TRBJ2.3 chains, already described in the past by other groups [6,23,24,25,26,27]. These public TCRs are expressed by CD8+ T cell clonotypes expanded in the blood and synovial fluid of B*27-positive patients with AS and in the blood and aqueous humour of patients with acute anterior uveitis (AAU) [6]. Interestingly, Hanson et al. have found an enrichment of CD8+ T cell clonotypes, mainly recognizing epitopes from herpesviruses such as the Epstein–Barr virus (EBV) and Cytomegalovirus (CMV), in B*27-positive patients with AS compared to matched healthy subjects [27]. Accordingly, we found CD8+ T cells specific for an EBV-derived epitope, pEBNA3A (RPPIFIRRL), in B*27:05 carriers, mostly affected by AS, but not in B*27:09-positive individuals [28,29]. This EBV-derived peptide does not possess the classical B*27 consensus sequence but was previously described as immunodominant in the context of HLA-B*07 restriction [28,29,30,31]. Molecular dynamic simulations and experimental data explained such atypical presentation by the shift of the peptide into the B*27 binding cleft with the accommodation of ArgP1 into the B pocket, leaving the A pocket empty [28,29].

In this work, we show that ERAP1 and two haplotypes influence the occurrence of B*27:05-restricted CD8+ T lymphocytes towards this EBV-derived suboptimal peptide. In addition, we found that the B*27:05 peptidome might be extended to other epitopes sharing the RP motif at the N-terminus, including the self-peptide pDYNEIN (RPPIFGDFL). Taken together, these observations, sustained by computational data, renew the possible involvement of CD8+ T cells in AS chronic inflammation. In particular, environmental factors (i.e., latent viral infections) and a predisposing genetic background (HLA-B*27 and specific ERAP1 and two variants) might synergistically promote T cell responses with a possible relevance for the disease autoimmune/autoinflammatory processes.

## 2. Results

### 2.1. An AS-Protective ERAP1/ERAP2 Haplotype Negatively Correlates with the Ability of B*27:05 Carriers to Elicit a Specific CD8+ T Response towards the Suboptimal pEBNA3A Viral Peptide

**Figure 1:**
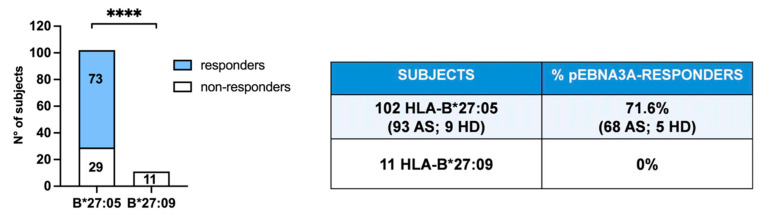
B*27:05- but not B*27:09-positive carriers possess pEBNA3A-specific CD8+ T cells. CD8+ T cell activation upon pEBNA3A stimulation was detected by IFNγ production and analysed in 102 B*27:05 carriers (93 patients with ankylosing spondylitis, indicated as AS, and 9 healthy donors, indicated as HD) and in 11 B*27:09 healthy donors. Of B*27:05 carriers, 71.6%, but no B*27:09 individuals, showed reactivity against pEBNA3A antigen (**** *p* value < 0.0001 calculated by Fisher’s two-tailed exact test).

Recently, we have disclosed the allele-specific capability of B*27:05, but not of B*27:09 molecules to present an EBV-derived antigen (RPPIFIRRL) from pEBNA3A that induced the activation of specific CD8+ T cells despite lacking the proper B*27 consensus motif [28,29]. Notably, this is a well-known immunodominant antigen in the context of the HLA-B*07 restriction [30,31]. We have enrolled new B*27:05 individuals, both AS patients and healthy donors and B*27:09 healthy subjects, thus confirming our previous observations demonstrating that 71.6% of B*27:05 individuals were responsive to this peptide, whereas no reactivity occurred in the B*27:09 donors (Figure 1).

**Figure 2:**
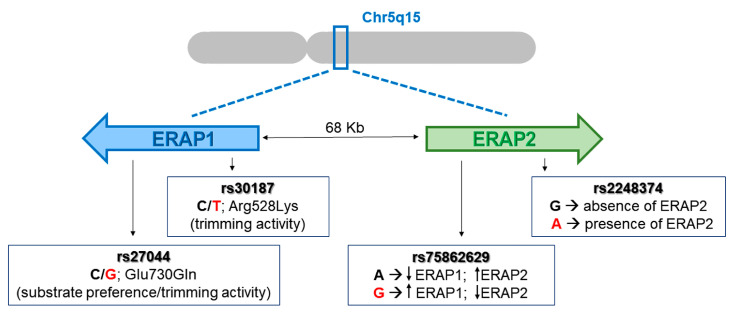
Gene organization (5q15) of ERAP1 and ERAP2 and the SNPs analysed. The cartoon shows ERAP1 and ERAP2 genes that are oppositely oriented on the 5q15 chromosome. In red are reported the AS-risk variants for each SNP.

Considering that the estimated EBV incidence in the global population is 90–95%, such CD8+ T cell response, although very frequent in B*27:05 subjects, did not cover all the expected EBV-seropositive individuals [32]. Thus, to exclude the possibility that the pEBNA3A non-responders could be EBV-negative donors, we performed an EBV serological test confirming the positivity in 100% of cases (unresponsive B*27:05 and B*27:09 donors). Hence, the lack of such a viral response should be explained by some other mechanisms. Considering the pivotal role of ERAP1 and ERAP2 in shaping the HLA class I ligandome, we hypothesized that specific ERAP1/ERAP2 polymorphisms could influence this atypical CD8+ T cell response. To this end, we analysed four SNPs of ERAP1 and ERAP2 (rs27044; rs30187; rs75862629; rs2248374) shown to be associated with the susceptibility to AS (Figure 2) [7,8,13,33,34].

The genotype analysis of the B*27:05 carriers, 73 pEBNA3A responders and 29 non-responders, pointed out that the AS protective variants, especially those of ERAP1, were more frequent in the cohort of pEBNA3A non-responders (both AS patients and HD), although a significant difference was found only for the rs30187 polymorphism (Table 1). Notably, the C allelic variant was more represented in the non-responders group when evaluating either the allelic or the genotype frequency (CC vs. CT + TT) (* *p* value = 0.037 and *** *p* value = 0.0007, respectively) (Table 1).

Moreover, taking into account the combination of the four SNPs, we identified the ERAP1/ERAP2 haplotype CCAG (rs27044-rs30187-rs75862629-rs2248374) as the more frequent in the cohort of pEBNA3A non-responders compared to the pEBNA3A responders (* *p* value= 0.0179) (Table 2). Interestingly, such a combination, described as protective for AS, implies a lower amount of ERAP1 with moderate enzymatic activity together with the absence of ERAP2, suggesting that this genotype affects the quantity of pEBNA3A and the probability of B*27:05 molecules to load this suboptimal 9mer into the Endoplasmic Reticulum (ER) [12,13,34].

### 2.2. Peptides with the RP Motif at the N-Terminus Trigger CD8+ T Cell Reactivity in the B*27:05 Context of Presentation

**Figure 3:**
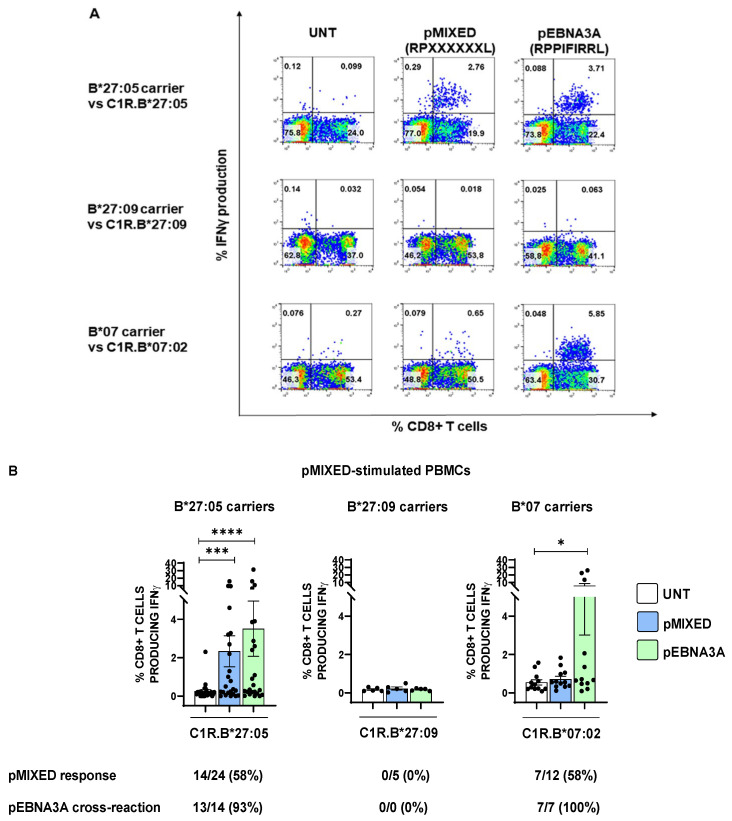
The B*27:05 allele induces a relevant activation of CD8+ T cells by presenting a mix of peptides with RP at the N-terminus. (**A**) Representative IFNγ-producing CD8+ T cells in PBMCs stimulated with pMIXED and re-activated with pMIXED or pEBNA3A. The panels are representative of the results obtained in 1 out of 24 B*27:05 subjects (left), in 1 out of 5 B*27:09 subjects (middle) and in 1 out of 12 B*07 subjects (right). (**B**) CD8+ T cell responses to pMIXED stimulation by B*27:05, B*27:09 and B*07 cohorts and cross-reactivity to pEBNA3A. Unexpectedly, the highest reactivity to pMIXED was found in B*27:05 subjects (21 AS patients, 3 HD) (left), whereas there was only a weak response in the B*07 context (2 AS patients, 2 PsA patients, 2 RA patients and 6 HD) (right). As expected, pMIXED did not induce CD8+ T cell activation in B*27:09 carriers (middle). The subjects were arbitrarily considered as responders when the percentage of IFNγ-producing CD8+ T cells was ≥0.1 (expressed as the difference between the response towards pMIXED or pEBNA3A pre-pulsed APC with that of APC incubated with the medium alone). In B*27:05 carriers, the recall stimulations either with pMIXED- or pEBNA3A-pulsed C1R.B*27:05 cells produced a statistically significant increase of IFNγ-producing CD8+ T cells compared to the stimulations by unpulsed cells (*** *p* value < 0.001 and **** *p* value < 0.0001, respectively). On the contrary, in the case of B*07 individuals, only the recall stimulation with pEBNA3A yielded a statistically significant difference (* *p* value < 0.05). Friedman test; **** *p* value < 0.0001; *** *p* value < 0.001.

The X-Pro bonds at the N-terminus are poorly cleaved by both ERAP1 and ERAP2 aminopeptidases, suggesting that a high trimming activity of the ERAP1/ERAP2 combination could promote an enrichment of suboptimal HLA-B*27 peptides featuring the N-terminal RP motif [35,36,37,38]. Such consideration implies that, aside from pEBNA3A, the AS-risk ERAP1 and ERAP2 variants could favour a higher relative proportion of other epitopes with a similar N-terminal sequence (RP), suitable for the B*27:05 antigen presentation with consequent CD8+ T cell responses. In line with this hypothesis, we stimulated the peripheral blood mononuclear cells (PBMCs) of 24 B*27:05 carriers (21 AS patients and 3 HD) for 14 days with a cocktail of peptides, hereinafter reported as pMIXED, possessing the RPXXXXXXL sequence, wherein the X could be each of the 20 amino acids, in equal ratio. In parallel, we extended such analysis to five B*27:09-positive carriers to exclude that this allele might share the degeneracy of B*27:05 in terms of “mis-peptidome”. Additionally, since the pMIXED peptides technically possessed the optimal HLA-B*07 consensus sequence, with the Pro in P2 and the Leu in PΩ, we evaluated the CD8+ T cell responses in 12 HLA-B*07 subjects (6 HD, 2 patients with AS, 2 with psoriatic arthritis (PsA) and 2 with rheumatoid arthritis (RA)) [39,40]. As expected, none of the five B*27:09 subjects displayed T cell responses upon stimulation by pMIXED (Figure 3A,B). Interestingly, the stimulation of PBMCs with this cocktail of peptides promoted a strong CD8+ T activation in HLA-B*27:05 subjects (*** *p* value < 0.001), whereas the magnitude of the response was modest in B*07 carriers (*p* value = ns). Nevertheless, the frequency of the pMIXED response was the same in the two cohorts (58%) (Figure 3B). Interestingly, in the B*07 group, all CD8+ T cell responses induced by pMIXED were cross-reactive to pEBNA3A (7 out of 7) (* *p* value < 0.05), with a higher amplitude in comparison to that in B*27:05 carriers (3.5% and 5.7%, respectively), although this result was strongly influenced by 3 out of 7 B*07 subjects. These results might suggest that the HLA-B*07 prevalently binds and presents pEBNA3A antigen within the pMIXED pool of peptides. Presumably, for the B*27:05 cohort, other suboptimal ligands with the RPXXXXXXL sequence can be recognized by TCRs evoking a detectable T cell activation (*** *p* value < 0.001) as demonstrated by one case of a pMIXED-driven CD8+ T cell response totally independent from pEBNA3A and several cases in which the magnitude of activation was higher with the former than the latter peptide. Nevertheless, also in the B*27:05 context, the CD8+ T cell responses raised against pMIXED were mostly cross-reactive to pEBNA3A (93% of cases).

### 2.3. pEBNA3A-Responsive CD8+ T Cells Are Cross-Reactive against a Self-Peptide from Dynein Motor Protein

**Figure 4:**
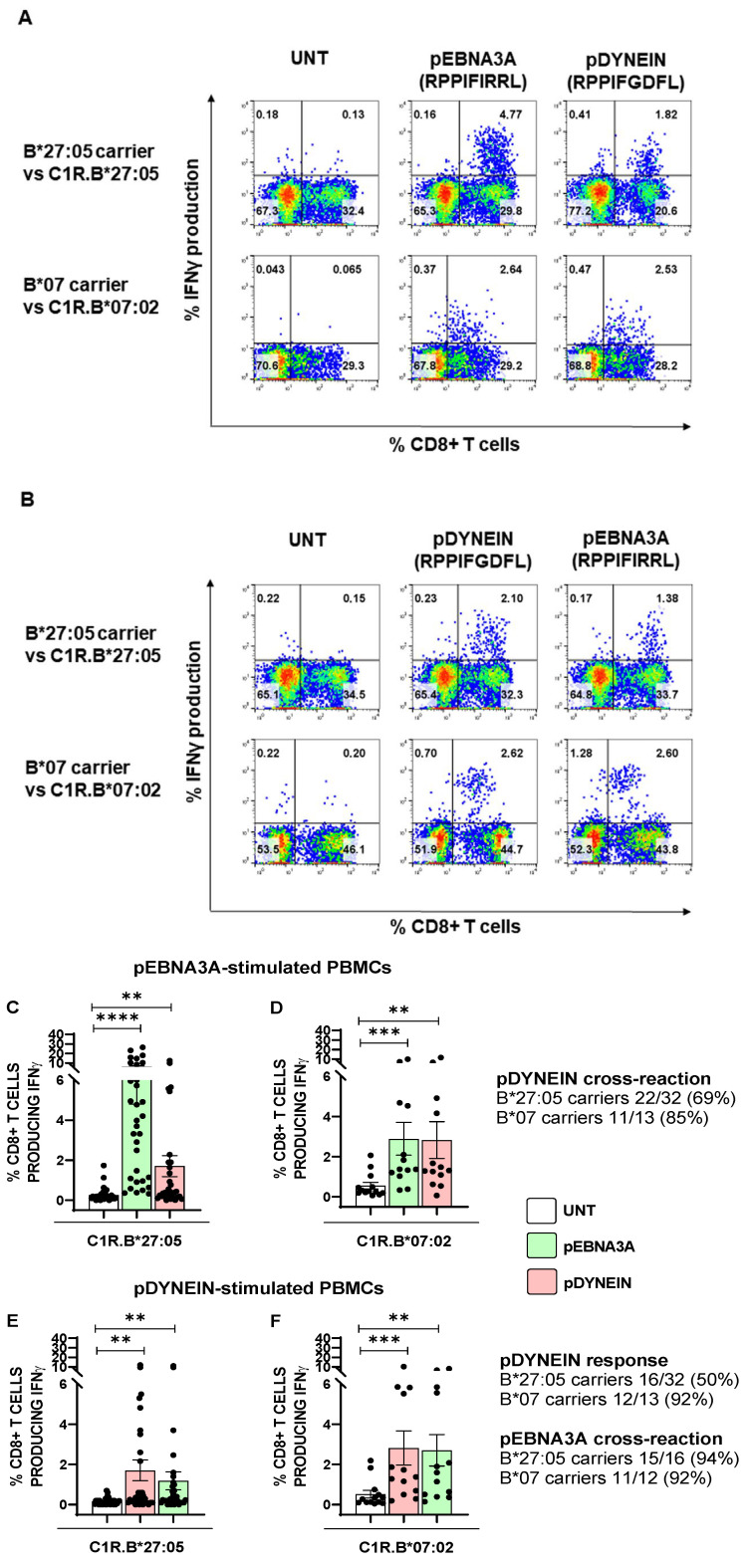
pEBNA3A-responsive CD8+ T cells are cross-reactive against a self-peptide. IFNγ production by CD8+ T cells from PBMCs stimulated with pEBNA3A (**A**) or pDYNEIN (**B**) in one representative subject out of 32 B*2705 carriers (29 AS patients, 3 HD) (**A**,**B** upper panels) and in 1 representative subject out of 13 B*07 carriers (4 AS patients, 2 PsA patients, 2 RA patients and 5 HD) (**A**,**B** lower panels). The cross-reactivity to pDYNEIN (**A**) and pEBNA3A (**B**) is also shown. The magnitude of pEBNA3A CD8+ T cell response was higher in B*27:05 subjects (**C**) than in B*07 carriers (**D**) and the cross-reactivity to pDYNEIN was found in both contexts, although at higher frequency in the B*07 background. The stimulation with pDYNEIN in B*27:05 (**E**) compared to B*07 (**F**) subjects produced a more frequent response in the latter group (50% vs. 92%), whereas the percentage of cross-reactive CD8+ T cell responses to pEBNA3A was high in both cases (94% vs. 92%). We arbitrarily considered the subjects as responders when the percentage of IFNγ-producing CD8+ T cells (expressed as the difference between the response to peptide as pre-pulsed APC with APC incubated with the medium alone) was ≥ 0.1. Friedman test; **** *p* value < 0.0001; *** *p* value < 0.001; ** *p* value < 0.005.

Our data demonstrated that, in contrast to the B*27:09 allele, B*27:05 can load peptides with an atypical fitting into the binding cleft, eliciting a detectable CD8+ T cell activation. These data prompted us to assess whether such suboptimal epitopes could encompass self-peptides as well. By performing a blast analysis in the data bank resource (https://blast.ncbi.nlm.nih.gov/Blast.cgi (accessed on 1 July 2023)), we found a self-peptide derived from dynein axonemal heavy chain 2, named pDYNEIN (2771–2779 RPPIFGDFL), with a sequence identical to pEBNA3A except for P6-P8. In order to assess a possible molecular mimicry between these two epitopes, we stimulated PBMCs from 32 B*27:05 carriers responsive to pEBNA3A (both AS patients and HD), 5 B*27:09 subjects and 13 B*07 carriers responsive to pEBNA3A (both patients and controls) with pEBNA3A (20 µg/mL) or pDYNEIN (40 µg/mL) for 14 days and tested the reciprocal CD8+ T cell cross-reactivity. As expected, B*27:09 carriers, who did not exhibit any pEBNA3A reaction, were also lacking the response towards the self-peptide. Despite the fact that the pEBNA3A should be immunodominant in the context of HLA-B*07 restriction and suboptimal for B*27:05 molecules, the magnitude of CD8+ T cell responses was higher in the B*27:05 than in HLA-B*07 carriers (Figure 4A,C,D). In fact, the average percentage of IFNγ production by CD8+ T cells was 6% in B*27:05 individuals and 2.9% in B*07 subjects (Figure 4A). By contrast, the cross-reaction to pDYNEIN, albeit present in both HLA-B contexts, was more relevant in B*07 (8 patients with immune mediated diseases and 5 HD) compared to B*27:05 (29 patients with AS and 3 HD) carriers, as evidenced by both the percentage of IFNγ-producing T cells (average: 2.8% and 1.7%, respectively) and the frequency (85% and 69%, respectively) (Figure 4A,C,D). Moreover, while the B*07 subjects elicited a comparable CD8+ T cell response to either pEBNA3A or pDYNEIN as first stimulus, the pDYNEIN induced less intense CD8+ T cell responses in B*27:05 subjects (Figure 4B,E,F). Accordingly, we found a lower frequency of pDYNEIN response in B*27:05 compared to B*07 (50% and 92%, respectively) with a different percentage of IFNγ-producing CD8+ T cells (average: 1.7% and 2.8%, respectively). Moreover, such pDYNEIN-responsive cells were extensively cross-reactive to pEBNA3A in both B*27:05 and B*07 contexts (94% and 92%, respectively), with a different magnitude (average: 1.2% and 2.7%, respectively) (Figure 4B,E,F).

### 2.4. Peptides with the RP Motif at the N-Terminus Do Not Stabilize the B*27 Molecules

**Figure 5:**
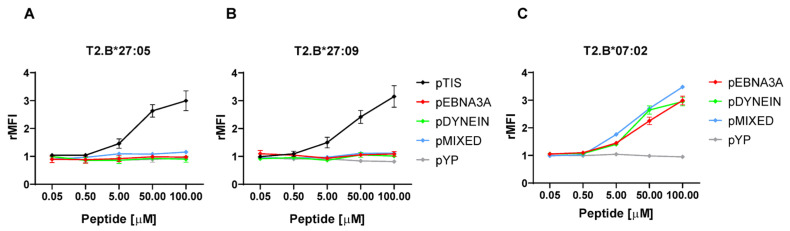
Peptides with the RP motif at the N-terminus are unable to stabilize B*27 molecules. Staining of B*27:05 (**A**), B*27:09 (**B**) or B*07:02 (**C**) molecules stably expressed on T2 transfectants performed by using ME1 mAb after cell incubation with the indicated peptide concentrations. As for B*27 molecules, pTIS (RRLPIFSRL) was used as a positive reference. Additionally, an irrelevant peptide (pYP) was used as a negative control for HLA-B alleles. Results are expressed as relative mean fluorescence intensity (rMFI) obtained with peptide-pulsed compared to unpulsed T2 cells. Values represent the mean ± SEM of three independent experiments.

The capability of the B*27:05, but not of B*27:09 molecule to present a suboptimal ligand evoking a specific CD8+ T cell response might be due to its intrinsic higher flexibility rather than a higher stability of the complexes [28,29]. Consistently with our previous pEBNA3A binding data obtained by using T2 cells stably expressing HLA-B*27:05 or -B*27:09 alleles [28,29], we did not observe surface stabilization mediated by pMIXED or pDYNEIN nor differences between the two B*27 alleles (Figure 5A,B). On the other hand, pEBNA3A, pDYNEIN and pMIXED, having an optimal B*07 binding motif, well stabilized these molecules on T2 cells (Figure 5C). Nevertheless, pMIXED, albeit inducing stable B*07:02 complexes (Figure 5C), turned out to be unable to induce vigorous CD8+ T responses (Figure 3A,B).

### 2.5. Molecular Dynamic (MD) Simulations of HLA-B Subtypes

**Figure 6:**
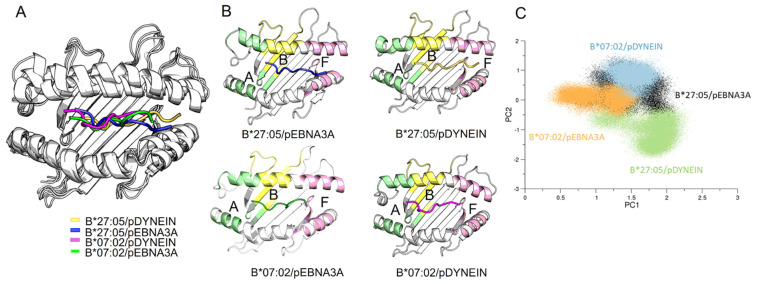
Binding groove and peptide conformations. (**A**) Superimposition of the averaged structures of the binding grooves for the systems in the study. (**B**) The averaged structures are displayed separately, and the binding groove pockets are shown with A pocket in green, B pocket in yellow and F pocket in pink. (**C**) Principal component analysis of the binding grooves.

To find a structural clue about the functional T cell data obtained with the viral peptide in comparison to the self-peptide in the context of the two HLA-B alleles, we performed computational analyses. MD simulations of HLA-B*27:05 and HLA-B*07:02 alleles bound to pEBNA3A and pDYNEIN (Figure 6A,B and Figure S1) were carried out to characterize their structural and dynamical behaviours. Two replicas of about 500 ns were performed for each complex.

To compare the motions of each system, a principal component analysis computed on the alpha carbon of the binding grooves (aa residue 1–180) was performed (Figure 6C). Our data showed that both B*27:05 and B*07:02 binding grooves explored similar conformations during the dynamics, except for the B*27:05/pDYNEIN complex. Mapping the interactions established between the viral and self-peptides and the binding grooves (Table 3), our results showed that, as expected, pEBNA3A and pDYNEIN formed a higher number of hydrogen bonds in complex with B*07:02, consistently with their proper B*07 binding consensus motif.

Looking at the peptide placement into the binding grooves (Table 4), the disposition of the peptides seemed to be more influenced by the peptide sequence than the binding groove. Thus, regardless of the HLA-B subtype, the P1 residue in the pDYNEIN was closer to the A pocket. Notably, the residue P9 in the B*07:02/pEBNA3A complex resulted well located in the pocket F, in line with the hydrogen bonds analysis.

Furthermore, pDYNEIN showed a lower solvent exposure compared to pEBNA3A in both HLA-B subtypes (Table 5).

Interestingly, the root-mean-square fluctuation (RMSF) analysis showed a different behaviour of the pEBNA3A peptide when bound to the HLA-B*07:02 compared to B*27:05 (Figure S2). In fact, the B*07:02 fluctuations were very limited with respect to the other simulated systems (Figure S2). This is in line with the entropy analysis (Table 6), which showed large and negative values of the difference between pEBNA3A and pDYNEIN bound to HLA-B*07:02 or, even, between pEBNA3A bound to HLA-B*07:02 compared to B*27:05. Furthermore, the contact analysis displayed a high fluctuation of both viral and self-peptides in complex with the B*27:05 (Figure S3A) and a higher fluctuation of the pDYNEIN with respect to pEBNA3A bound to HLA-B*07:02, resulting in a larger number of binding groove residues in contact with the former peptide (Figure S3B). These results suggest that, although both peptides remain in the binding groove along the MD simulations, the nature of their interaction with the HLA-B*07:02 subtype is rather different.

## 3. Discussion

In this study, we have highlighted how AS-risk variants of ERAP1 and 2 cooperate with the disease-associated HLA-B*27:05 subtype, unlike the non-associated B*27:09 allele, in the processing and presentation pathway of suboptimal viral antigens, which unleash CD8+ T cell responses in B*27:05 carriers. This is a relevant finding given the double implication of HLA-B*27 in the immune-mediated disorders, particularly spondyloarthritis, as well as in viral protection, recently extended to SARS-CoV-2 [3,33,41,42].

The suboptimal EBV-derived antigen pEBNA3A (RPPIFIRRL), here investigated, has been already described by our group as an uncanonical poor ligand of HLA-B*27 molecules, which fits in the binding cleft, leaving the A pocket empty, as suggested by both functional and computational evidence (Figure 6 and Figure S1) [28,29]. Nevertheless, pEBNA3A, which is a well-known immunodominant epitope in the context of HLA-B*07 presentation, has also turned out to be a highly immunogenic B*27:05 antigen able to induce CD8+ T cell activation in 71.6% of B*27:05-positive carriers (73.1% of patients with AS and 55.6% of healthy subjects) (Figure 1) in spite of being a suboptimal ligand [30,31,43]. Notably, the magnitude of the CD8+ T cell response upon pEBNA3A stimulation was higher in the HLA-B*27:05 than in the HLA-B*07 subjects (Figure 4C). In addition, double-B*27:05/B*07-positive AS patients displayed a better T cell reactivity against this viral antigen in the context of B*27:05 compared to B*07 molecules in two out of three cases ([28]). Thus, pEBNA3A is undoubtedly a highly efficient antigen when presented by the B*27:05 allele, although theoretical predictive algorithms and experimental binding assays assigned it as a non-B*27 binder (Figure 5A). What remains mechanistically unclear is the lack of pEBNA3A reactivity in the B*27:09 background [28,29].

However, although EBV seropositive, a certain number of HLA-B*27:05 subjects (28.4%) remained immunologically unresponsive to pEBNA3A. This evidence prompted us to look for other factors or mechanisms influencing the processing and presentation of such an uncanonical antigen. We hypothesized that this allele-dependent “misbehaviour” could be critically influenced by the peptide handling in the ER made by ERAP1 and ERAP2 [10,44]. In fact, ERAP1 is virtually able to cleave all N-terminal residues, except for Pro, with a particular efficacy on hydrophobic residues, whereas ERAP2 preferentially targets the basic ones [33,35,36,37,38]. This suggests that a suboptimal B*27 peptidome, encompassing peptides with N-terminal sequences similar to pEBNA3A, could be favoured by a high trimming efficacy of ERAP1 and ERAP2 variants, also associated with AS susceptibility [12,33]. Moreover, the well-documented epistasis between HLA-B*27 and ERAP1 variants in affecting the AS risk sustains the convergence of these two genes in the antigen presentation pathway with consequent effects on T cell immunity [45,46]. In line with these observations, we performed a genetic analysis of B*27:05 subjects, responsive and unresponsive to pEBNA3A, to assess whether specific ERAP1 and 2 allelic variants and/or haplotypes correlated with the ability to mount the pEBNA3A-specific CD8+ T response. Focusing on two ERAP1 (rs27044 and rs30187) and two ERAP2 (rs75862629 and rs2248374) SNPs, described as AS associated, we found a trend of more frequent protective variants in the pEBNA3A not-responding cohort, with statistical significance reached for the rs30187 SNP (Table 1). Interestingly, among the haplotypes resulting from the combination of the four SNPs, we found the CCAG (rs27044/rs30187/rs75862629/rs2248374) haplotype, protective for AS, more significantly represented in the pEBNA3A non-responders group (Table 2) [12,13,33,34]. This could imply that, in B*27:05 carriers, the same genetic allelic variants of ERAP1 and ERAP2 not predisposing to autoimmunity do not favour the presentation of uncanonical viral peptides as well.

AS belongs to the so-called “MHC-I-opathies”, together with the psoriasis, Behçet’s disease, birdshot uveitis and acute anterior uveitis for which GWAS have highlighted the association to ERAP1 and, sometimes, ERAP2, apart from the involvement of a key specific HLA-class I gene [47]. Notably, an ERAP1 risk haplotype for psoriasis, including the same variants analysed in this study, has been proven to efficiently generate a melanocyte-derived autoantigen with a parallel increase of the predisposing HLA-C*06:02 molecules [48]. This finding makes ERAP1 a candidate to be a promising target of therapeutic approaches in psoriasis and, hopefully, in the related MHC-I-associated diseases.

A further important question was whether the immunologically productive presentation of pEBNA3A, having the unusual N-terminal RP motif, was a sporadic case, or if the B*27:05 degeneracy was more frequent than anticipated. This was an important issue to address, since the peptides with proline at P2 are spared by ERAP1 and 2 and the AS-risk haplotypes of ERAP1 and 2, having a high trimming activity, should increase their relative proportion in the pool of ER peptides [35,46]. To this end, PBMCs of HLA-B*27 and HLA-B*07 carriers have been stimulated with a cocktail of nonamers (pMIXED) sharing with pEBNA3A both the N-terminal RP motif and the leucine at P9 (RPXXXXXXL). Notably, HLA-B*27:05 subjects, mostly patients with AS, raised a vigorous CD8+ T cell response towards such suboptimal mixed peptides (Figure 3A,B). Certainly, within the pMIXED, there was a sufficient amount of pEBNA3A peptide to allow the expansion of pEBNA3A-driven CD8+ T cells (Figure 3A,B). Nevertheless, the response to pMIXED in the B*27:05 background should be also triggered by other unknown peptides, since some pMIXED-stimulated CD8+ T cells were not or were only slightly cross-reactive to pEBNA3A. Therefore, it can be speculated that, apart from pEBNA3A, other peptides within pMIXED, regardless of their theoretical unsuitability for B*27:05, as also shown by the binding data (Figure 5A), succeed in the interaction with the TCRs. Noteably, these peptides probably compete with each other for the B*27:05 loading and/or TCR recognition, resulting in a less intense CD8+ T cell activation after the recall stimulation with pMIXED compared to pEBNA3A (Figure 3A,B). A comprehensive TCR repertoire analysis of pMIXED- versus pEBNA3A-stimulated CD8+ T lymphocytes from B*27:05 carriers might be informative about the T cell poly- or oligoclonality of such responses. By contrast, the B*27:09 allele, being presumably less promiscuous and more flexible than the B*27:05 allele, selectively binds antigens with the canonical B*27 sequence, justifying the lack of response towards both pEBNA3A and pMIXED (Figure 3A,B) [29]. Although pMIXED completely fulfilled the HLA-B*07 binding motif, in the HLA-B*07 carriers, the pMIXED stimulation activated few CD8+ T lymphocytes that were extensively and strongly cross-reactive to pEBNA3A (Figure 3A,B). These data are difficult to interpret, but the optimal binding of pMIXED to the HLA-B*07 molecules, as also demonstrated by the stabilization assay (Figure 5C), allows us to speculate that the peptide cocktail contains strong ligands able to compete with each other, limiting the general expansion of CD8+ T cells specific for unknown peptides as well as for pEBNA3A. Conversely, the recall stimulation with the single pEBNA3A antigen enables a full response of specific CD8+ T cells in a context of no peptide competition (Figure 3A,B).

The putative extension of the B*27:05 ligandome to suboptimal peptides could be one of the explanations for the supremacy of such alleles in conferring good protection against viral infections [33,42]. The downside of the coin could be the presentation of self-peptides. Accordingly, using the data bank and searching for peptides with pEBNA3A-similar sequences allowed us to identify a self-peptide, pDYNEIN, with a pEBNA3A-identical sequence, except for P6-P8 residues.

In B*27:05 carriers, either AS patients or healthy controls, pEBNA3A-driven CD8+ T cells were found to be cross-reactive against pDYNEIN (69% of cases) (Figure 4C), and the direct stimulation with this self-peptide evoked T cell activation in 50% of B*27:05 subjects with 92% of pEBNA3A cross-reactivity (Figure 4E). Nevertheless, the implication of these pDYNEIN-driven or cross-reactive CD8+ T lymphocytes in the pathogenesis of AS is questioned by their occurrence in B*27:05 healthy donors as well as in the context of B*07 antigen presentation. Moreover, CD8+ T cell activation generated by the recognition of this self-peptide in association with the HLA-B*07 molecules appeared particularly robust either in terms of cross-reaction with pEBNA3A (Figure 4D) or upon direct peptide stimulation (Figure 4F). The more vigorous T cell functional effect of the pDYNEIN in complex with the HLA-B*07 in comparison to B*27:05 molecules can be explained by a better fitting of this peptide in the binding cleft of the former allele as supported by experimental binding results (Figure 5A,C) and computational data. Indeed, MD simulations suggested that such a behaviour can be due to the different interaction pattern between pDYNEIN and the binding grooves of the two HLA-B alleles (Figure 6A–C and Figure S3 and Table 3, Table 4, Table 5 and Table 6).

Nevertheless, in patients with AS, the capability of B*27:05 to promote CD8+ T responses cross-reactive towards self-peptides, when primarily induced by suboptimal viral antigens, could contribute to the spread and maintenance of the inflammation. Notably, this is not the first case of molecular mimicry between an EBV-derived peptide and a self-epitope displayed by B*27:05 molecules in patients with AS, supporting the pathogenic relevance of B*27:05 as an antigen-presenting molecule to CD8+ T cells [5,49]. Although there is no evidence of an EBV implication in the pathogenesis of AS, the persistence of the infection could not only modulate the T cell response, inducing a premature immune senescence of the cells, but could also worsen the inflammation by directing the effector functions of these cells, primarily activated by viral antigens, towards similar self-epitopes [50]. Accordingly, a relevant expansion of EBV- or CMV-directed CD8+ T cells in patients affected by AS compared to healthy subjects has been found [27]. This scenario could be reminiscent of the cross-reaction recently observed between microbial and self-antigens, favoured by the dysbiosis observed in AS patients, which sustains the autoimmune component of AS [6,21,22].

In conclusion, this work shows that the high flexibility of the binding cleft makes the B*27:05 allele capable of loading suboptimal viral antigens, which resulted highly immunogenic, mostly in the AS patient cohort, thus activating cross-reactive CD8+ T cell responses against self-antigens. In addition, this is the first evidence of a link between specific ERAP1/2 haplotypes and the occurrence of suboptimal viral-specific B*27-restricted CD8+ T cell responses in B*27:05 carriers, paving the way for experimental models useful to set new therapeutic strategies targeting such enzymes or, even, specific T clonotypes.

## 4. Materials and Methods

### 4.1. Study Subjects

A total of 102 HLA-B*27:05-positive subjects (93 AS-patients and 9 HD), 13 HLA-B*07-positive carriers (4 AS patients, 2 PsA patients, 2 RA patients and 5 HD) and 11 HLA-B*27:09-positive controls were enrolled in this study. Diagnosis of AS has been made according to modified New York criteria [51]. Subjects have been diagnosed with PsA according to the 2006 Classification of Psoriatic Arthritis (CASPAR) criteria, while the RA patients fulfilled the 2010 American College of Rheumatology (ACR) criteria [52,53]. Patients and controls were recruited at the Rheumatology Units of Sapienza University of Rome (Policlinico Umberto I, Roma and Ospedale S.M. Goretti, Latina) and at the Rheumatology Unit of Cagliari (Azienda Ospedaliero-Universitaria, Cagliari). The expression of HLA-B*27 and HLA-B*07 was determined by serological analysis using ME1 mAb (specificity: HLA-B*27; -B*07; -B*42; -B*67; -B*73 and -B*w22) or using an anti-human HLA-B*07-specific mAb (Sony Biotechnology, Weybridge, Surrey, UK), respectively. The HLA-B*27 subtypes were assessed through genomic analysis by using the Micro SSP Allele-specific HLA class I DNA typing tray B*27 (ONE LAMBDA, Thermo Fisher, Waltham, MA, USA) according to the manufacturer’s instructions. The study received the approval of the Ethics Committees of the University of Cagliari (PG/2018/16312) and Sapienza University of Rome (0018614/2019 and 6893/2022). All subjects provided written informed consent prior to the enrolment.

### 4.2. ERAPs Genotyping

Genomic DNA was obtained from EDTA-treated peripheral blood samples using the QIAamp DNA Blood Mini Kit (Qiagen, Hilden, Germany). SNP genotyping was performed by quantitative RT-PCR with a functionally tested TaqMan Allelic Discrimination Assay (rs30187: C_3056885_10; rs27044: C_3956870_10; rs2248374: C_25649529_10; 7300 Real-Time PCR System, Applied Biosystems, Foster City, CA, USA). SNP rs75862629 was directly sequenced by BioFab Research laboratories and A or G variants were discriminated by chromatogram analysis through BioEdit 7.2 software.

### 4.3. EVB Seropositivity

The sera were maintained at −80 °C immediately after blood withdrawals. Diluted sera were used for the detection of IgG antibodies to EVB-VCA by using the EVB-VCA IgG ELISA kit (MyBioSource, Huntingdon, UK) according to the manufacturer’s instructions.

### 4.4. Synthetic Peptides

pEBNA3A (RPPIFIRRL; aa 379–387), an immunodominant EBV epitope restricted by HLA-B*07; and pDYNEIN (RPPIFGDFL; aa 2689–2697), a self-peptide derived from dynein axonemal heavy chain 2 were used in this study [30,31]. We also used a cocktail of peptides, pMIXED, with the following sequence: RPXXXXXXL, where the X stands for each of the 20 amino acids in equal ratio. For the binding assay, a self-peptide restricted by HLA-B*27 (pTIS, RRLPIFSRL; aa 325−333) and an immunodominant HLA-B*35-restricted EBV epitope (YPLHEQHGM; aa 458−466) were included [28]. Peptides (purity > 95%) were purchased from Aurogene (Rome, Italy).

### 4.5. Cell Lines

TAP-defective CEM 174.T2 cells (ATCC number: CRL-1992™) and HMy2.C1R cells (ATCC number: CRL-1993™) stably expressing B*27:05, B*27:09 or B*07 were used [28]. Cells were cultured in heat-inactivated 10% fetal bovine serum (FBS; Euroclone Spa, Pero, Milan, Italy)/RPMI 1640 medium (Euroclone Spa, Pero, Milan, Italy) supplemented with 2 mmol/L L-glutamine, 100 U/mL penicillin, 100 μg/mL streptomycin. T2 and C1R stable transfectants were maintained in medium with the addition of G418 (800 μg/mL) or hygromycin B (200 μg/mL) (Euroclone Spa, Pero, Milan, Italy), respectively.

### 4.6. PBMCs Stimulation

PBMCs (4 × 10^6^) separated from whole blood by density gradient separation (Cedarlane Laboratories Ltd., Burlington, ON, Canada) were incubated with the pEBNA3A (20 μg/mL), pMIXED (20 μg/mL) or pDYNEIN (40 μg/mL) and cultured for 14 days at 2 × 10^6^ cells/mL in 5% FBS/RPMI complete medium and 10 U/mL of human recombinant interleukin 2 (IL2) (Roche Applied Science, Penzberg, Germany). On days 3, 9 and 12, fresh medium containing 20 U/mL of IL2 was replenished. After 14 days from the antigen stimulation, PBMCs were subjected to intracellular IFNγ staining.

### 4.7. Intracellular IFNγ Staining

Briefly, C1R transfectants were incubated overnight with pEBNA3A (40 μg/mL), pMIXED (40 μg/mL) or pDYNEIN (60 μg/mL) or with medium alone and thoroughly washed before being plated with antigen-stimulated PBMCs at a 5:1 PBMCs/APC ratio. After 30′, the cells were treated with brefeldin A (10 μg/mL) at 37 °C for 16 h. Cells were stained with the anti-CD8-FITC mAb (BioLegend, San Diego, CA, USA), 20′ on ice, fixed with 4% paraformaldehyde, 20′ on ice, permeabilized with 1% BSA/0.1% saponin in PBS 1X for 5′ at RT. Finally, cells were stained by an anti-IFNγ-APC mAb (BioLegend, San Diego, CA, USA) for 20′ at RT. The samples were acquired by a FACSCalibur flow cytometer (Becton Dickinson, Franklin Lakes, NJ, USA) and analysed by FlowJo 10 software (Tree Star Inc., Ashland, OR, USA). We arbitrarily considered the subjects as responders when the percentage of IFNγ-producing CD8+ T cells (expressed as the difference between the response of peptide pre-pulsed APC versus that of APC incubated with the medium alone) was ≥0.1.

### 4.8. T2 Stabilization Assay

The capability of the selected peptides to stabilize the B*27:05, B*27:09 or B*07 molecules on the cell surface of T2 stable transfectants was assessed according to the procedure previously described by Tedeschi et al. [28]. The surface amount of B*27 or B*07 molecules on T2 transfectants was determined by using ME1 mAb and a rabbit anti-mouse IgG-FITC (Jackson ImmunoResearch Europe, Suffolk, UK) as a secondary antibody. An antibody of the IgG isotype was also employed as a control. The results are shown as the mean ± SD of three independent experiments.

### 4.9. Statistics

Genotype analysis of the ERAP1 and ERAP2 SNPs and haplotypes between pEBNA3A responders and non-responders was performed by Haploview 4.2 software (http://www.broad.mit.edu/mpg/haploview/ (accessed on 1 July 2023); Cambridge, MA, USA) using the chi-square test. The pEBNA3A response in B*2705- or B*2709-positive subjects was compared by Fisher’s two-tailed exact test. Data obtained by IFNγ-production assays from pMIXED-, pEBNA3A- or pDYNEIN-stimulated PBMCs from B*27:05-, B*27:09- or B*07-positive carriers were compared by Friedman test. A *p* value < 0.05 was considered statistically significant.

### 4.10. MD Simulation

The TIP3P water model was used to solvate the systems [54]. A physiological concentration (0.15 M) of Na + Cl^−^ was added to neutralize the systems during the solvation. An energy minimization step was performed using the steepest descent algorithm without position restraints [55]. After the minimization, an NVT equilibration was performed using the V-rescaling thermostat and a temperature of 310K [56]. The box was resized to get the correct value of density. Finally, a production run of 500 ns was performed for each system in duplicate, using the same simulation setting. The electrostatic interactions were computed using the particle mesh Ewald method with a cut-off of 1.2 nm [57]. A cut-off of 1.2 nm was used for the van der Waals interactions. The simulations were performed using a CHARMM-36 force field, and the Gromacs Software version 2021.5 (University of Groningen, Groningen, The Netherlands) [55,58].

### 4.11. Principal Component Analysis

A principal component analysis was performed on the alpha carbon of the combined trajectories [59]. The aim of this analysis was to compare the motions of the proteins projecting the conformations explored by each system in the same space. In the graph, each dot represents an averaged conformation explored by the protein. Several dots form a cloud of conformation that represents the protein living space. Superimposition of the clouds indicates similarity between conformations, and thus similarity between protein motions. The analysis was performed using the gmx covar and gmx anaeig tools of Gromacs 2021.5 [55].

### 4.12. Solvent Exposure, Hydrogen Bonds, Peptides Displacement and the Contact Matrix

The averaged solvent exposure of the peptides was computed using the gmx sasa tool of Gromacs 2021.5 [55]. Hydrogen bonds were computed using the gmx hbond tool of Gromacs 2021.5 [56]. The peptide placement was computed using gmx mindist. Here, the distance was computed between the center of mass (COM) of each peptide residue and the COM of the pockets. The contact matrix was computed using the MDanalysis.analysis.distances.distance_array module provided by the MDAnalysis package [60]. The distances were computed on the alpha carbon of the binding grooves (aa residue 1–180) and the peptides.

## Figures and Tables

**Table 1:** Genotype distribution and allele frequency of ERAP1 and ERAP2 SNPs in B*27:05-positive pEBNA3A responders versus non-responders.

		pEBNA3A Responders (N = 73)		pEBNA3A Non-Responders (N = 29)		
	**Genotype**	**N**	**(%)**	**N**	**(%)**	
**ERAP1 rs27044 C/G Glu730Gln**	**CC**	20	27.4	12	41.4	
	**GC**	42	57.5	15	51.7	
	**GG**	11	15.1	2	6.9	
	**Allele**					
	**C**	82	56.2	39	67.2	
	**G**	64	43.8	19	32.8	
	**Genotype**	**N**	**(%)**	**N**	**(%)**	
**ERAP1 rs30187** **C/T Arg528Lys**	**CC**	7	9.6	11	37.9	*** p value 0.0007 *(CC* vs. *TT + CT)*
	**CT**	48	65.7	12	41.4	
	**TT**	18	24.7	6	20.7	
	**Allele**					
	**C**	62	42.5	34	58.6	* p value 0.037
	**T**	84	57.5	24	41.4	
	**Genotype**	**N**	**(%)**	**N**	**(%)**	
**ERAP2 rs75862629intergenic region** **A/G**	**AA**	58	79.5	25	86.2	
	**AG**	13	17.8	4	13.8	
	**GG**	2	2.7	0	0.0	
	**Allele**					
	**A**	129	88.4	54	93.1	
	**G**	17	11.6	4	6.9	
	**Genotype**	**N**	**(%)**	**N**	**(%)**	
**ERAP2 rs2248374** **G-> absence** **A** **-> presence**	**GG**	17	23.3	7	24.1	
	**AG**	43	58.9	20	69.0	
	**AA**	13	17.8	2	6.9	
	**Allele**					
	**G**	77	52.7	34	58.6	
	**A**	69	47.3	24	41.4	

The allelic and genotype frequencies for each SNP in B*27:05-positive pEBNA3A responders versus pEBNA3A non-responders are reported. The AS-risk variants are indicated in red. The C variant for rs30187 is more frequent in the pEBNA3A non-responders, both as allelic (* *p* value = 0.037) and genotype frequency (CC vs. CT + TT) (*** *p* value = 0.0007). Chi-square test; * *p* value < 0.05; *** *p* value < 0.001.

**Table 2:** ERAP1/ERAP2 haplotype distribution in B*27:05-positive pEBNA3A responders versus non-responders.

(rs27044-rs30187-rs75862629-rs2248374) Haplotypes	Frequency	pEBNA3A- Responders	pEBNA3A-Non-Responders	χ^2^	*p* Value
**G-T-A-G**	0.261	0.271	0.235	0.267	0.6052
**C-C-A-A**	0.238	0.221	0.281	0.844	0.3582
**C-C-A-G**	**0.182**	**0.141**	**0.283**	**5.604**	* 0.0179
**C-T-A-A**	0.078	0.092	0.042	1.458	0.2272
**G-T-G-A**	0.065	0.071	0.050	0.309	0.5782
**C-T-A-G**	0.058	0.063	0.047	0.177	0.6736
**G-T-A-A**	0.057	0.064	0.038	0.554	0.4567
**C-C-G-G**	0.027	0.031	0.017	0.283	0.5947
**G-C-A-G**	0.017	0.022	0.003	0.910	0.3401
**C-T-G-A**	0.011	0.015	0.002	0.624	0.4295

Analysis of the ERAP1/ERAP2 haplotype frequency in B*27:05-positive pEBNA3A responders versus non-responders identifies the CCAG haplotype as the most represented in the latter group (* *p* value = 0.0179). rs27044 C/**G**, rs30187 C/**T**, rs75862629 A/**G** and rs2248374 **A**/G. In bold, the AS-risk alleles. Haplotype analysis was performed by Haploview 4.2 software; chi-square test; * *p* value < 0.05.

**Table 3:** Hydrogen bonds established between the binding grooves and the peptide residues in each simulation.

Groove B*27:05/B*07:02	pDYNEIN/ HLA-B*27:05	pEBNA3A/ HLA-B*27:05	pDYNEIN/ HLA-B*07:02	pEBNA3A/ HLA-B*07:02
Glu 45/Glu 45		P1		P1
Tyr 59/Tyr 59			P1	
Glu 63/Asn 63	P1	P1	P1	
Lys 70/Gln 70				P7
Thr 73/Thr 73				P8
Asp 77/Ser 77				
Thr 80/Asn 80				P9
Asn 97/Ser 97				P7
Tyr 99/Tyr 99		P1		
His114/Asp 114				P7
Ile 142/Ile 142				
Lys 146/Lys 146	P7–P9		P8	P8–P9
Trp 147/Trp 147				
Leu 156/Arg 156				P7
Tyr 159/Tyr 159	P1	P1	P1	
Glu 163/Glu 163	P1		P4	

The residues of the peptides are reported as P1 (for the first residue) to P9 (for the last one).

**Table 4:** Peptide placement in the binding grooves.

HLA/Peptide Complex	P1—A Pocket Distance (nm)	P1—B Pocket Distance (nm)	P9—F Pocket Distance (nm)
B*27:05/pDYNEIN	0.36 ± 0.04	0.46 ± 0.03	1.23 ± 0.14
B*27:05/pEBNA3A	0.42 ± 0.05	0.39 ± 0.04	0.8 ± 0.03
B*07:02/pDYNEIN	0.35 ± 0.04	0.63 ± 0.07	1.024 ± 0.04
B*07:02/pEBNA3A	0.45 ± 0.04	0.45 ± 0.04	0.34 ± 0.04

**Table 5:** Solvent exposure of the peptides.

HLA/Peptide Complex	Solvent Exposure (nm^2^)
B*27:05/pDYNEIN	14.5 ± 0.3
B*27:05/pEBNA3A	16.4 ± 0.1
B*07:02/pDYNEIN	14.4 ± 0.7
B*07:02/pEBNA3A	16.6 ± 0.5

**Table 6:** Entropies of the peptides.

Complexes	TΔS (KJ/mol)
B*27:05/pEBNA3A-pDYNEIN	−11.16 ± 5
B*07:02/pEBNA3A-pDYNEIN	−50.5 ± 6.2
B*07:02-B*27:05/pDYNEIN	−5.8 ± 17.9
B*07:02-B*27:05/pEBNA3A	−44.98 ± 18.9

## Data Availability

The data presented on this study are available on request from the corresponding authors.

## Supplementary Materials

The following supporting information can be downloaded at: https://www.mdpi.com/article/10.3390/ijms241713335/s1.
