# Supplementary material for: ERAP1 and ERAP2 Haplotypes Influence Suboptimal HLA-B*27:05-Restricted Anti-Viral CD8+ T Cell Responses Cross-Reactive to Self-Epitopes
Source: Int J Mol Sci. 2023 Aug 28;24(17):13335. doi: 10.3390/ijms241713335 (PMC10488187; doi:10.3390/ijms241713335)
Supplement: Supplementary file 1 [file ijms-24-13335-s001.zip › ijms-2556567-supplementary.pdf]

Supplementary materials

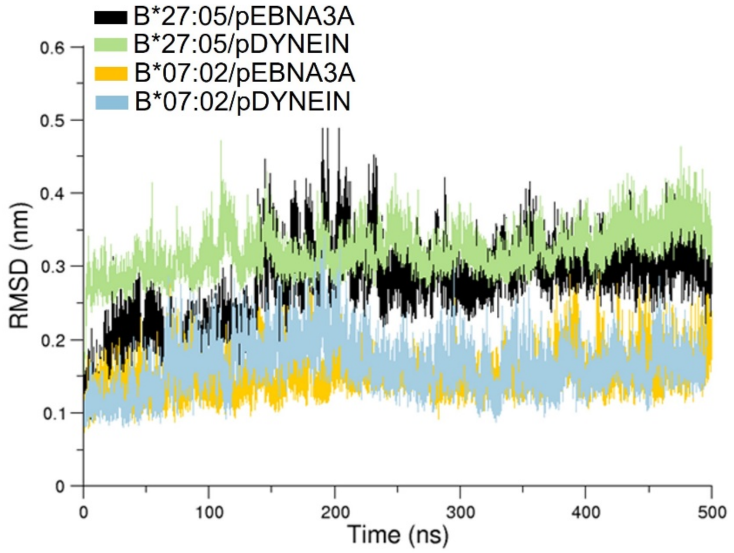

Figure S1. RMSDs computed on the alpha carbons of the entire system. The analysis shows that the B*27:05 subtype deviates more from the initial structure with respect to the B*07:02 along the MD trajectory.

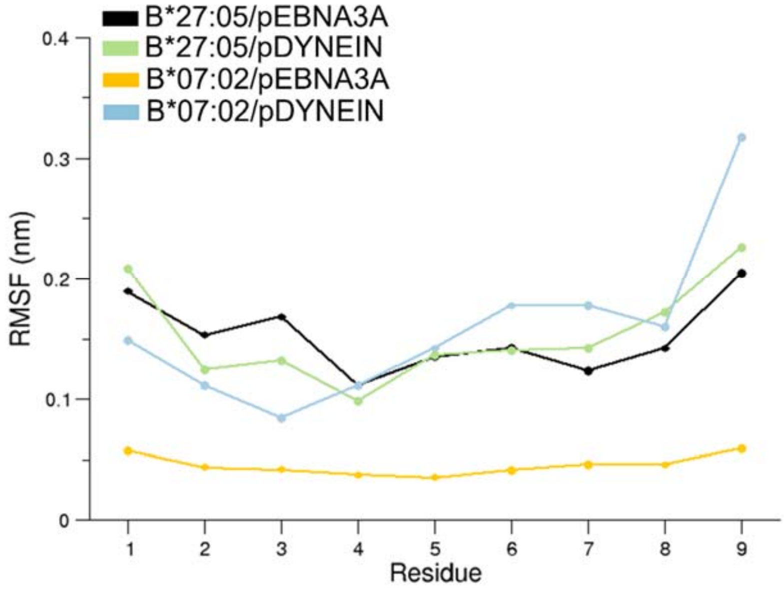

Figure S2. RMSF computed on the backbone of the peptides. The analysis shows very limited fluctuations of the pEBNA3A peptide when it binds to B*07:02 with respect to the other simulated systems.

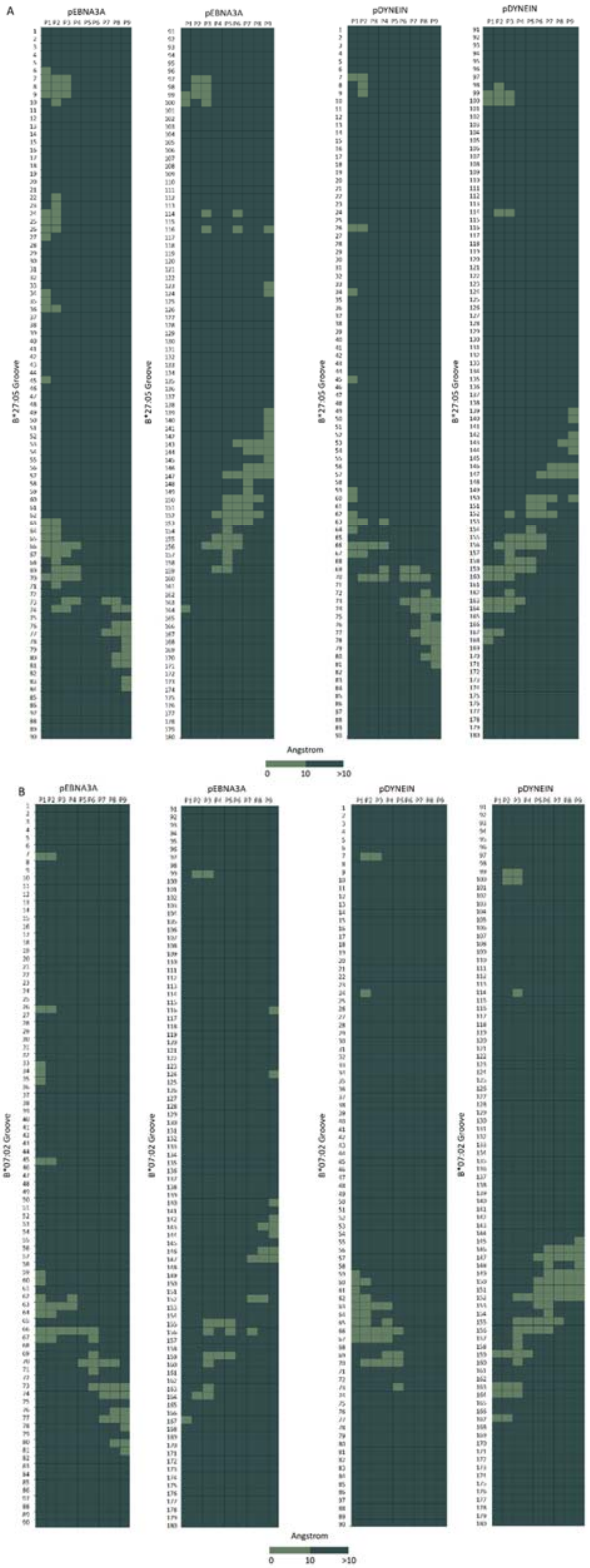

Figure S3. Contact matrices of B*27:05 and B*07:02 The distances between the alpha carbon of the peptides and the binding grooves (aa residues 1-180) are reported as matrices. (A), the matrices computed for B*27:05 in complex with pEBNA3A (on the left) and pDYNEIN (on the right). (B), the matrices computed for B*07:02 in complex with pEBNA3A (on the left) and pDYNEIN (on the right). For each system, the binding groove was split into two parts (aa residues 1-90 and 91-180), thus producing two matrices, for a better visualization. Light-green squares indicate a distance below/equal to 10 Å. Dark-green squares indicate a distance greater than 10 Å. Although the regions of interactions are quite conserved in all the simulated systems, the pDYNEIN establishes interactions with a higher number of residues of the binding grooves of both B*27:05 and B*07:02, underlining its elevated fluctuation.
